# Potential Role of Hsp70 and Activated NK Cells for Prediction of Prognosis in Glioblastoma Patients

**DOI:** 10.3389/fmolb.2021.669366

**Published:** 2021-05-17

**Authors:** Dominik Lobinger, Jens Gempt, Wolfgang Sievert, Melanie Barz, Sven Schmitt, Huyen Thie Nguyen, Stefan Stangl, Caroline Werner, Fei Wang, Zhiyuan Wu, Hengyi Fan, Hannah Zanth, Maxim Shevtsov, Mathias Pilz, Isabelle Riederer, Melissa Schwab, Jürgen Schlegel, Gabriele Multhoff

**Affiliations:** ^1^ Department of Radiation Oncology, School of Medicine, Technical University Munich (TUM), Munich, Germany; ^2^ Central Institute for Translational Cancer Research, School of Medicine, Technical University Munich, Munich, Germany; ^3^ Department of Neurosurgery, School of Medicine, Technical University Munich (TUM), School of Medicine, Munich, Germany; ^4^ Institute of Cytology of the Russian Academy of Sciences, St. Petersburg, Russia; ^5^ Department of Neuroradiology, School of Medicine, Technical University Munich (TUM), Munich, Germany; ^6^ Department of Neuropathology, Technical University Munich (TUM), Munich, Germany

**Keywords:** Hsp70, biomarker, glioblastoma, NK cells, tumor immunology, predcition of prognosis

## Abstract

Despite rapid progress in the treatment of many cancers, glioblastoma remains a devastating disease with dismal prognosis. The aim of this study was to identify chaperone- and immune-related biomarkers to improve prediction of outcome in glioblastoma. Depending on its intra- or extracellular localization the major stress-inducible heat shock protein 70 (Hsp70) fulfills different tasks. In the cytosol Hsp70 interferes with pro-apoptotic signaling pathways and thereby protects tumor cells from programmed cell death. Extracellular Hsp70 together with pro-inflammatory cytokines are reported to stimulate the expression of activatory NK cell receptors, recognizing highly aggressive human tumor cells that present Hsp70 on their cell surface. Therefore, intra-, extracellular and membrane-bound Hsp70 levels were assessed in gliomas together with activatory NK cell receptors. All gliomas were found to be membrane Hsp70-positive and high grade gliomas more frequently show an overexpression of Hsp70 in the nucleus and cytosol. Significantly elevated extracellular Hsp70 levels are detected in glioblastomas with large necrotic areas. Overall survival (OS) is more favorable in patients with low Hsp70 serum levels indicating that a high Hsp70 expression is associated with an unfavorable prognosis. The data provide a first hint that elevated frequencies of activated NK cells at diagnosis might be associated with a better clinical outcome.

## Introduction

Heat shock protein 70 (Hsp70), the major stress-inducible member of the 70-kDa heat shock protein family is evolutionary conserved and ubiquitously expressed in nearly all subcellular compartments (Multhoff, 2007; Radons, 2016). Under physiological conditions Hsp70 maintains protein homeostasis by preventing protein aggregation, supporting the folding of nascent polypeptides and transporting other proteins across membranes (Mayer and Bukau, 2005). Stress such as hyperthermia, ischemia, nutrient deficiency or therapeutic interventions strongly upregulate the synthesis of Hsp70. Cytosolic Hsp70 interferes with signaling pathways that affect apoptosis, proliferation and differentiation (Mayer and Bukau, 2005), and as a result, elevated intracellular Hsp70 levels protect tumor cells from apoptotic cell death (Beere, 2005). Due to their high metabolic demand many aggressive tumor types exhibit an overexpression of Hsp70 in the cytosol which promotes tumorigenesis (Calderwood et al., 2006). Following stress, Hsp70 rapidly translocates into the nucleus and/or interacts with lysosomal membranes to stabilize them (Nylandsted et al., 2004). In highly aggressive non-small lung cell cancer (NSCLC) Hsp70 is presented on the plasma membrane and gets released into the extracellular milieu (Gunther et al., 2015). We have previously shown that serum levels of Hsp70 correlate with the viable tumor mass and affect peripheral blood lymphocyte (PBL) compositions and profiles in patients with NSCLC (Gunther et al., 2015). Extracellular Hsp70 exists in the form of a free protein which predominantly originates from dying tumor cells or is bound to exosomes that are actively released by viable tumor cells (Multhoff et al., 1999; Gastpar et al., 2005).

As part of the innate immune system NK cells are responsible for the first line of defense against infectious diseases and cancer. NK cells release T cell recruiting chemokines including IFN-γ, TNF-α and NK cell cytotoxicity is mediated via granzyme B/perforin, cell death receptor interactions and/or antibody dependent cellular cytotoxicity (ADCC) through low affinity Fc-γ receptors. NK cell activity is independent of a clonal T cell receptor and major histocompatibility complex antigens (MHC), but is tightly regulated by a fine balance between the expression density of activating receptors with a short immunoreceptor tyrosine-based activation motif (ITAM) and inhibiting receptors with a long immunoreceptor tyrosine-based inhibitory motif (ITIM) (Pockley et al., 2020). To license NK cells to kill membrane Hsp70+ (mHsp70+) tumor cells they can be activated by an incubation with full length Hsp70 protein, Hsp70-expressing exosomes (Multhoff et al., 1999; Gastpar et al., 2005) or a 14-mer Hsp70-peptide TKD (TKDNNLLGRFELSG) derived from the C-terminal substrate binding domain in combination with interleukin 2 (IL-2) (Multhoff et al., 1997; Multhoff et al., 2001). Due to the smaller size, favorable biodistribution, higher purity and easier GMP-production the Hsp70-peptide TKD is advantageous to recombinant Hsp70 protein for the stimulation of NK cells. A pilot (Kokowski et al., 2019) and clinical phase I study (Krause et al., 2004) demonstrated excellent safety profiles of autologous, *ex vivo* TKD/IL-2-activated NK cells, and patients with advanced NSCLC showed promising clinical responses in a randomized clinical phase II trial after adoptive transfer of *ex vivo* TKD/IL-2-activated NK cells (Multhoff et al., 2020).

Apart from a study in grade IV glioblastoma/astrocytoma patients (Thorsteinsdottir et al., 2017) insight into the expression and localization of Hsp70 in different types of brain tumors and its impact on the prevalence and activity of NK cells is limited. Therefore, intracellular, extracellular and mHsp70 levels were profiled as potential prognostic biomarkers and stimuli for NK cells in different gliomas.

Gliomas predominantly originate in the brain parenchyma. Among the diffuse growing gliomas which are classified according to the WHO classification as grade II, III and IV gliomas, glioblastoma is the most common primary malignant tumor of the central nervous system in adults (Ostrom et al., 2014; Ellingson et al., 2015). Glioblastoma is treated with surgery, radiation and a temozolomide-based chemotherapy (Stupp et al., 2005). However, despite this multimodal therapy these patients still have a dismal prognosis with median survival rates of approximately 15 months (Stupp et al., 2005). Tumor grading, age, isocitrate-dehydrogenase 1 (IDH1) and methylation status of the DNA repair gene O-6-methylguanin-DNA methyltransferase (MGMT) promotor, performance status and extent of resection are considered for predicting clinical outcome (Kita et al., 2009; Weller et al., 2017). However, presently only the MGMT promotor status qualifies for the prediction of clinical responses to temozolomide.

The goal of the present study was to identify chaperone- and immune-related biomarkers in the circulation of glioma patients for a better prediction of outcome in glioblastoma patients.

## Materials and Methods

### Patients

The study was approved by the local ethical committee of the medical faculty of the Technical University Munich (TUM, #2403/09) and was conducted in accordance with the Declaration of Helsinki. Written informed consent was obtained from all patients before the start of therapy. EDTA-anticoagulated blood (for flow cytometry) and non-coagulated blood (for ELISA) was collected from patients with different types of brain tumors attending in the Department of Neurosurgery. EDTA-blood samples (7.5 ml) were taken form a total of 88 patients with brain tumors. According to the WHO classification of Tumors of the Central Nervous System (Louis et al., 2016) and histopathological analysis (Dpt. Neuropathology), 2 patients were diagnosed as having grade II oligodendroglioma, 6 as having grade II astrocytoma, 8 as having grade III anaplastic oligodendroglioma, 6 as having grade III anaplastic astrocytoma, and 66 patients as having grade IV gliomas. The samples were reviewed for this study. All patients were treated with radiotherapy concomitant with temozolomide after surgery according to the Stupp-regimen (Stupp et al., 2005). Clinical outcome and extracellular Hsp70 levels below and above a cut-off value of 3.5 ng/ml which was determined by using the Youden-index, at diagnosis were associated with OS in grade IV glioblastoma patients (*n* = 34) by Kaplan–Meier analysis.

### Tumor Volumetry by Magnetic Resonance Imaging (MRI)

Pre-operative MRI scans were performed on a 3Tesla MRI scanner (Philips Achieva, Philips Ingenia (Philips Medical Systems, Netherlands B.V.) or Siemens Verio (Siemens Healthcare, Erlangen, Germany) and analyzed as 3DT2-FLAIR and 3DT1 pre- and post-contrast sequences. Tumor volumes were determined by 2 independent neuroradiologists. MRI sequences (3DT2-FLAIR, 3DT1 post-contrast) were co-registered using a 3D Slicer (www.slicer.org) (Fedorov et al., 2012) and tumor volumes were segmented semi-automatically using a freely available software ITK-SNAP (www.itksnap.org) (Yushkevich et al., 2006) in contrast-enhancing and FLAIR-hyperintense tumor areas.

### Immunohistochemistry (IHC) and Scoring of Hsp70 in Brain Tumor Sections

A total of 24 formalin-fixed paraffin-embedded (FFPE) sections (3 µm) of different brain tumors (Dpt. Neuropathology) and of brain tissues from non-tumor patients were stained for the expression of intracellular Hsp70 content. Briefly, the sections were dewaxed and hydrated in xylol (9713.3, Roth) and descending concentrations of ethanol (ethanol absolute, 2,7694, Fischar GmbH, ethanol 96% v/v, 2,7687, Fischar GmbH). After heating at 50–60°C for 30 min in citrate buffer (target retrieval solution, S1699, Dako, 1:10 dilution in ddH_2_O), sections were washed in ddH_2_O, H_2_O_2_ (3% v/v) and sodium azide (0.1% v/v) in phosphate buffered saline (PBS, D8537-500 ML, Sigma Life Science) to block the activity of endogenous peroxidase. Unspecific binding was blocked by rabbit serum (5% v/v in antibody diluent, S2022, Dako). The sections were incubated with the Hsp70 monoclonal antibody cmHsp70.1 (Stangl et al., 2011) (multimmune GmbH, Munich, Germany) or a respective IgG1 isotype-matched control antibody (1:500 in antibody diluent, S2022, Dako) at 4°C overnight. Antibody binding was detected by incubating horseradish peroxidase (HRP) labeled polymer conjugated with secondary antibodies (Envision + System HRP labeled polymer anti-mouse, K4001, Dako) for 20 min, followed by a Diaminobenzidine chromogen (DAB + substrate buffer, K3468, Dako, DAB + chromogen, K3468, Dako) for exactly 5 min and nucleus was counterstained with hematoxylin and eosin (T865.2, Roth). Sections were embedded and coverslipped in Eukitt quick-hardening mounting medium (Roti-Histokitt 100 ml, 6638.1, Roth). Staining of Hsp70-negative (fibroblasts) and Hsp70+ (FaDu tumor cells) was included in each immunohistochemical staining for a better inter-assay comparability of the staining patterns. To compare the staining intensities, the staining procedure was performed absolute identically with identical reagents. Sections were analyzed on an Axio Vison microscope (Carl Zeiss, Jena) using the software ImageScope version 12.3.2.8013. According to the gray scale intensity of the tumor tissue measured by ImageJ in arbitrary units (AU), the Hsp70 expression was classified into low and high intensity. Nuclear vs. nuclear + cytosolic Hsp70 localization was analyzed separately. Scoring of the Hsp70 staining was performed blindly and independently by 2 researchers and 2 neuropathologists.

### Flow Cytometric Analysis of mHsp70 Expression on Viable Brain Tumor Cells

Freshly aspirated tumor material (Dpt. Neurosurgery) was cut in 1 mm^3^ pieces, incubated with trypsin for 8 min and forced through a sterile mesh (70 µm strainer). After washing and resuspension of the single cell suspension in PBS/10% v/v FCS, 5 × 10^5^ cells per tube were incubated with the following fluorescence-labelled antibodies for 30 min on ice: tube 1: IgG1-FITC/APC (BD Biosciences), tube 2: CD45-APC (Thermo Fisher) and cmHsp70.1-FITC (multimmune GmbH), tube 3: pan-HLA class I-FITC (F5662, Sigma). After two washing steps, 7AAD (BD Biosciences) was added directly before flow cytometric analysis. Only viable, 7AAD-negative tumor cells which are CD45-negative (to exclude lymphocytes), were gated and analyzed on a FACSCalibur™ flow cytometer (BD Biosciences). The pan-HLA class I antibody staining served as a positive control and isotype-matched control antibodies were used as respective negative controls. A minimum of 100.000 events were recorded for each measurement.

### Measurement of Circulating Hsp70 Levels

Serum (S-Monovette 7.5 ml Z, Sarstedt, Nürnbrecht, Germany) was obtained after centrifugation of peripheral blood (10 min, 5000 rpm). Aliquots (300 μL) were stored at −80°C. Hsp70 serum concentrations were determined in duplicates using the DuoSet^®^ IC Human/Mouse/Rat Total Hsp70 ELISA (R&D Systems, Minneapolis, MN, United States) following the manufacturer’s protocol or using our “in-house” Hsp70 ELISA (Louis et al., 2016) which predominantly detects exosomal Hsp70 derived from viable tumor cells. Briefly, 96-well MaxiSorp Nunc-Immuno plates (Thermo, Rochester, NY, United States) were coated overnight with an Hsp70 specific antibody in sodium carbonate buffer (0.1 M sodium carbonate, 0.1 M sodium hydrogen carbonate, pH 9.6). The biotinylated mouse mAb cmHsp70.1 (multimmune, Munich, Germany) in HRP-Protector (Candor Bioscience GmbH, Wangen im Allgäu, Germany) serves as a detection antibody. After washing with phosphate buffered saline (PBS, Life Technologies, Carlsbad, CA, United States), unspecific binding was blocked with liquid plate sealer (Candor Bioscince GmbH, Wangen im Allgäu, Germany). Plasma samples, diluted in StabilZyme Select Stabilizer (Diarect GmbH, Freiburg im Breisgau, Germany) were incubated for 30 min at room temperature and after a washing step, horseradish peroxidase-conjugated streptavidin (Senova GmbH, Weimar, Germany), dissolved in HRP-Protector (Candor Bioscience GmbH, Wangen im Allgäu, Germany) was added. Colorimetric analysis using a substrate reagent (BioFX TMB Super Sensitive One Component HRP Microwell Substrate, Surmodics, Inc., Eden Prairie, MN, United States) was performed on a Microplate Reader (VICTOR X4 Multilabel Plate Reader, PerkinElmer, Waltham, MA, United States).

### Immune Phenotyping of Peripheral Blood Lymphocytes (PBLs)

Lymphocyte subpopulations in the peripheral blood were profiled by multicolor flow cytometry on a FACSCalibur™ instrument (BD Biosciences) using the following fluorescence-labelled antibodies: T cell antibodies CD3-PerCP (BD-345766), CD4-FITC (BD-555346), CD8-FITC/PE (BD-347313/BD-555366), NK cell antibodies CD56-FITC/APC (BD-345811/BD-555518), CD94-FITC (BD-555888), NKG2D-PE (FAB139P-R&D Systems), NKp30-PE (PNIM3709-Beckman Coulter), NKp46-PE (PNIM3711-Beckman Coulter), and B cell antibody CD19-PE (555413-BD Biosciences). All stainings included appropriate isotype- and fluorescence-matched control antibodies. After incubation (15 min) in the dark at room temperature, cells were washed in PBS/10% v/v FCS. Red blood cells were lyzed by incubating in BD FACS™ lysing solution (1:9 dilution in _dd_H_2_O, 349202-BD Biosciences, 10 min). In brief, lymphocytes were gated according to their FSC/SSC characteristics and doublets were excluded. A representative graph of the lymphocyte gating strategy is shown in Supplementary Figure S1. NK and T cells were discriminated based on their CD56 and CD3 expression together with additional NK and T cell markers, as mentioned above. Isotype-matched control antibodies were used as negative controls.

For regulatory T cell (Treg) analysis selected CD3^+^ (CD3-PerCP, BD-345766) T cells were subdivided into CD4^+^ (CD4-FITC, BD-555346) and CD8^+^ (CD8-FITC BD-347313) T cells and the percentage of CD25^+^ (CD25-APC, BD-340907) and FoxP3+ (FoxP3-PE, BD-560046) cells were determined in the CD4^+^ and CD8^+^ subpopulation after fixing (buffer A, 1:10 in _dd_H_2_O, 51–9005451-BD Biosciences) and permeabilizing the cells (buffer C, 1:50 in buffer A, 51–9005450-BD, 30 min). The percentage of positively stained cells was determined within a defined lymphocyte gate.

### *Ex vivo* Stimulation of Peripheral Blood Lymphocytes With Hsp70/IL-2 or Hsp70-peptide TKD/IL-2

To mimic potential immunostimulatory effects of circulating Hsp70 in glioblastoma patients, PBL of healthy individuals (*n* = 4) were stimulated either with Hsp70 or Hsp70-peptide TKD together with IL-2 (Novartis Pharma GmbH Nürnberg, 17152.00.00). EDTA-blood (7.5 ml) was collected from healthy volunteers with a median age of 23.5 (range: 22–24 years) from which PBL were separated by Ficoll density gradient centrifugation. After counting, trypan blue-negative, viable cells (5 × 10^6^/ml) were resuspended in RPMI-1640 with l-glutamine, 10% v/v FCS, penicillin G (100 IU/ml) and streptomycin (100 μg/ml) and stimulated either with recombinant Hsp70 (10 μg/ml) and IL-2 (100 IU/ml) or Hsp70-peptide TKD (2 μg/ml, TKDNNLLGRFELSG, GMP-grade, Bachem AG, Bubendorf) and IL-2 (100 IU/ml) for 5 days (Multhoff et al., 1999; Multhoff et al., 2001). After stimulation, cells were counted and 200.000 cells each were analyzed by flow cytometry using T and NK cell specific antibodies (T cell: CD3-PerCP, BD-345766; NK cell: CD56-APC, BD-555518; CD94-FITC, BD-555888; CD69-PE, BD-341652), shown above. Viability of PBL after Ficoll separation and after stimulation was always >95%.

### Statistics

The statistical analysis was performed using the programming language R, R studio version 3.5.2. Normal distribution was tested by the Shapiro–Wilk normality test. Parametric data were analyzed by ANOVA and post-hoc Tukey tests, non-parametric were analyzed by using the Kruskal Wallis test and Wilcoxon signed-rank test, as appropriate. A value of *p* < 0.05 was considered as representing statistically significant differences.

## Results

### Magnetic Resonance Imaging and Hsp70 Staining of Brain Tumors

**FIGURE 1:**
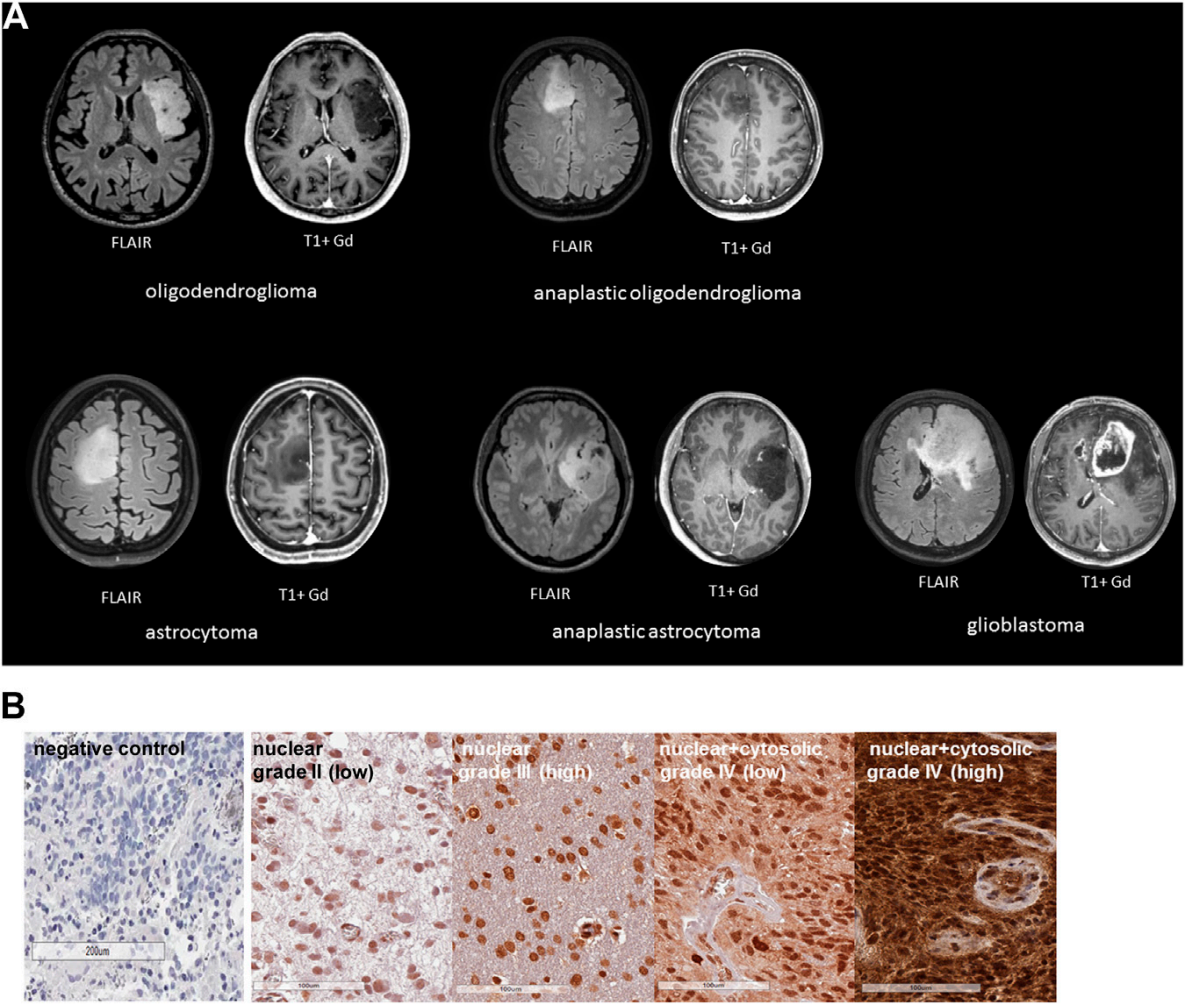
**(A)** Representative MR images of different glioma grades: oligodendroglioma, astrocytoma (grade II), anaplastic oligodendroglioma, anaplastic astrocytoma (grade III), glioblastoma (grade IV). Left, FLAIR images, right, T1 images with Gadolinium (Gd). **(B)** Representative immunohistochemical (IHC) stainings of Hsp70 in control tissue and tumor sections (3 µm) of grade II, III and IV gliomas. Shown are comparisons of nuclear vs. nuclear + cytosolic stainings patterns with low or high Hsp70 staining intensity.

Clinical characteristics such as WHO grade, age, diagnosis, isocitrate-dehydrogenase 1 (IDH-1) wild type (WT) of the glioma patients recruited into the study are summarized in Table 1. Representative MR images of different brain tumors are shown in Figure 1A. Low grade tumors typically do not show contrast enhancement or necrosis and appear hyperintense on FLAIR images and hypointense on T1-weighted images, whereas glioblastomas show a thick irregular contrast enhancement, central necrosis, and perifocal FLAIR-hyperintensity that represents tumor infiltration and edema. FFPE sections of tumor biopsies were stained immunohistochemically for their intracellular Hsp70 content. Only tumor areas, but not the surrounding tissue are considered for the evaluation. Figure 1B shows representative views of the different localizations (nuclear/nuclear + cytosolic) and staining intensities (low/high) of Hsp70 in control tissue, grade II, III and IV gliomas. Table 2 summarizes the data of grade II (*n* = 3), grade III (*n* = 4) and grade IV gliomas (*n* = 17) in a four-by-four analysis. In summary, 2 of 3 grade II gliomas (67%) were scored as Hsp70 low and 1 of 3 (33%) as high, 1 of 4 grade III gliomas (25%) were scored as Hsp70 low and 3 of 4 (75%) as high, and 5 of 17 grade IV gliomas (30%) were scored as Hsp70 low and 12 of 17 (70%) as high (Table 2). Hsp70 rapidly translocates into the nucleus upon stress. All lower grade gliomas showed only a nuclear Hsp70 localization, whereas 16 of 17 (94%) grade IV gliomas exhibited a nuclear + cytosolic Hsp70 localization (Table 2). In general the Hsp70 staining intensity is weaker in lower grade gliomas compared to glioblastomas. Due to the higher amount of Hsp70 in high grade gliomas, Hsp70 is found in the nucleus and cytosol of grade IV gliomas.

**TABLE 1:** WHO grade, age, diagnosis, isocitrate-dehydrogenase 1 (IDH-1) wild type (wt) of glioma patients.

WHO grade	Median age (years)	Age range (years)	Diagnosis	IDH-1 wt	*n* subgroups	*n* total
II	30.5	24–52	Oligodendroglioma	0	2	
			Astrocytoma	0	6	8
III	49.5	29–61	Anaplastic oligodendroglioma	0	8	
			Anaplastic astrocytoma	1	6	15
IV	62	21–89	Glioblastoma	65	66	66

**TABLE 2:** Correlation of glioma grading (grade II, III, IV) with the Hsp70 staining intensity (low vs. high) and Hsp70 localization (nuclear Vs. nuclear + cytosolic).

WHO grade	Hsp70 intensity low	Hsp70 intensity high
II	2/3 (67%)	1/3 (33%)
III	1/4 (25%)	3/4 (75%)
IV	5/17 (30%)	12/17 (70%)
WHO grade	Hsp70 localization nuclear	Hsp70 localization nuclear + cytosolic
II	3/3 (100%)	0/3 (0%)
III	4/4 (100%)	0/4 (0%)
IV	1/17 (6%)	16/17 (94%)

### Membrane Hsp70 (mHsp70) Expression on Viable Brain Tumor Cells

**FIGURE 2:**
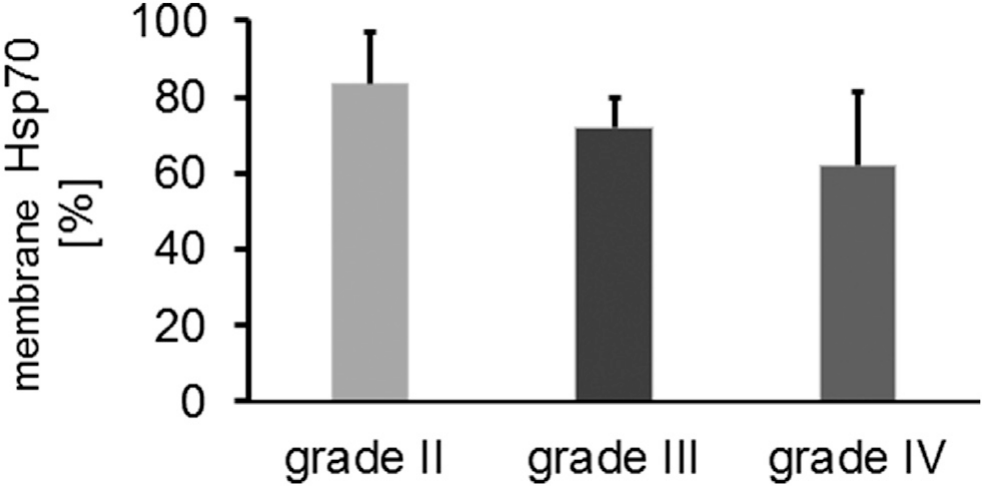
Comparative analysis of the proportion of mHsp70 + tumor cells in grade II (*n* = 3), grade III (*n* = 6) and grade IV (*n* = 28) gliomas (Kruskal Wallis; not significant, ns).

In addition to the cytosolic Hsp70 content, the mHsp70 positivity was determined in isolated, single cell suspensions of freshly resected brain tumors and control brain tissue by flow cytometry. Due to the poor viability of brain tumor cells *in vitro* (20–30%), the mHsp70 status could be determined only in a total of 37 glioma samples. An intact plasma membrane is key for determining the membrane status of Hsp70 because otherwise a false positive, cytosolic Hsp70 staining will be determined by flow cytometry. The mHsp70 staining intensity appeared to be slightly higher in glioblastomas compared to low grade gliomas. Overall the proportion of mHsp70+ tumor cells was above 60% in all glioma samples and did not differ significantly between the different tumor grades (Figure 2). Control brain tissue showed no mHsp70 positivity (data not shown).

### Extracellular Hsp70 Concentrations

**FIGURE 3:**
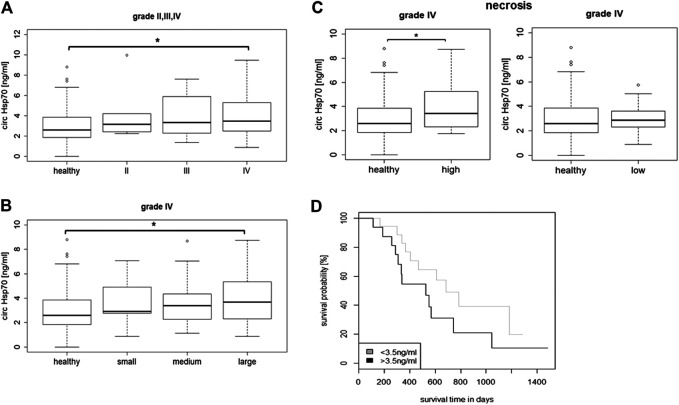
**(A)** Circulating Hsp70 concentrations in healthy controls (*n* = 150) vs. grade II (*n* = 5) grade III (*n* = 12) and grade IV (*n* = 51) glioma patients. **(B)** Circulating Hsp70 concentrations in healthy controls (*n* = 150) vs. grade IV glioma patients (*n* = 44) with small (<30 cm^3^, *n* = 6), medium (30–90 cm^3^, *n* = 16) and large (>90 cm^3^, *n* = 22) tumor volumes, as determined with an ELISA detecting free Hsp70 (Kurskal Wallis; *p < 0.05). **(C)** Circulating Hsp70 concentrations in healthy controls (*n* = 150) vs. grade IV glioma (*n* = 27) with a high (>10%, *n* = 13) and low proportion of necrosis (<10%, *n* = 14), as determined with an ELISA detecting free Hsp70 (Wilcoxon rank; *p < 0.05). **(D)** Kaplan-Meier analysis of the overall survival (OS) in glioblastoma patients with Hsp70 serum levels below (*n* = 16) and above (*n* = 18) a threshold of 3.5 ng/ml (Logrank; *p* = 0.1).

Serum Hsp70 levels were determined in grade II (*n* = 5), grade III (*n* = 12) and grade IV (*n* = 51) patients at first diagnosis using the R&D ELISA which predominantly detects free Hsp70 originating from dying tumor cells and by an “in-house” Hsp70 ELISA (Breuninger et al., 2014) that detects both, free and liposomal Hsp70. As shown in Figure 3A, the median free Hsp70 concentrations in the circulation of patients with grade IV gliomas at diagnosis were significantly higher (3.48 ng/ml) than in healthy controls (*n* = 150, 2.60 ng/ml, Tukey; *p < 0.05). Median serum Hsp70 concentrations in grade II and III glioma patients were 3.14 ng/ml and 3.34 ng/ml, respectively, but did not differ significantly to that of healthy controls (Figure 3A). With respect to liposomal Hsp70 which is representing the viable tumor mass in other tumor entities (Sharma and Allison, 2020), no significant differences were observed in all tumor grades (data not shown).

According to their tumor volumes, patients with grade IV gliomas (*n* = 44) were separated into groups of patients with small (<30 cm^3^), medium (30–90 cm^3^) and large (>90 cm^3^) tumors. The median tumor volumes in patients with small tumors (*n* = 6) was 9.2 cm^3^ (range 4.8–26 cm^3^), in those with medium tumors (n = 16) was 71.7 cm^3^ (range 35.3–81.8 cm^3^), and in those with large tumors (*n* = 22) was 140.9 cm^3^ (range 99–222 cm^3^). As shown in Figure 3B, glioblastoma patients with large tumor volumes had significantly higher median Hsp70 concentrations in the serum (3.68 ng/ml, *n* = 22) than healthy controls (2.60 ng/ml, *n* = 150) as calculated by the Kruskal Wallis test (*p < 0.05), whereas those patients with small and medium tumor volumes did not differ significantly from the healthy control group.

According to MRI, the subgroup of grade IV gliomas (n = 27) was subdivided into a group with a high proportion of necrosis (>10%, *n* = 13) and a group with a low proportion of necrosis (<10%, n = 14). The concentration of free Hsp70 in the circulation (3.42 ng/ml) in glioblastoma patients with highly necrotic tumors was significantly higher compared to healthy controls (2.60 ng/ml, Wilcoxon rank Test, *p < 0.05) (Figure 3C), whereas, that of patients with low tumor necrosis did not differ significantly to healthy controls (2.87 vs. 2.60 ng/ml) (Figure 3C). As expected, liposomal Hsp70 levels did not differ significantly in patients with low and high proportions of tumor necrosis, but were elevated compared to that of healthy controls (data not shown). A Kaplan-Meier analysis of glioblastoma patients whose free Hsp70 serum levels were either below (*n* = 18) or above (*n* = 16) a threshold of 3.5 ng/ml which distinguishes healthy human individuals from tumor patients (Breuninger et al., 2014) revealed a trend toward an improved OS in patients whose Hsp70 serum levels were below 3.5 ng/ml (*p* = 0.1), but did not reach statistical significance (Figure 3D).

### Composition of Lymphocyte Subpopulations in the Peripheral Blood of Patients With Different Brain Tumors

**FIGURE 4:**
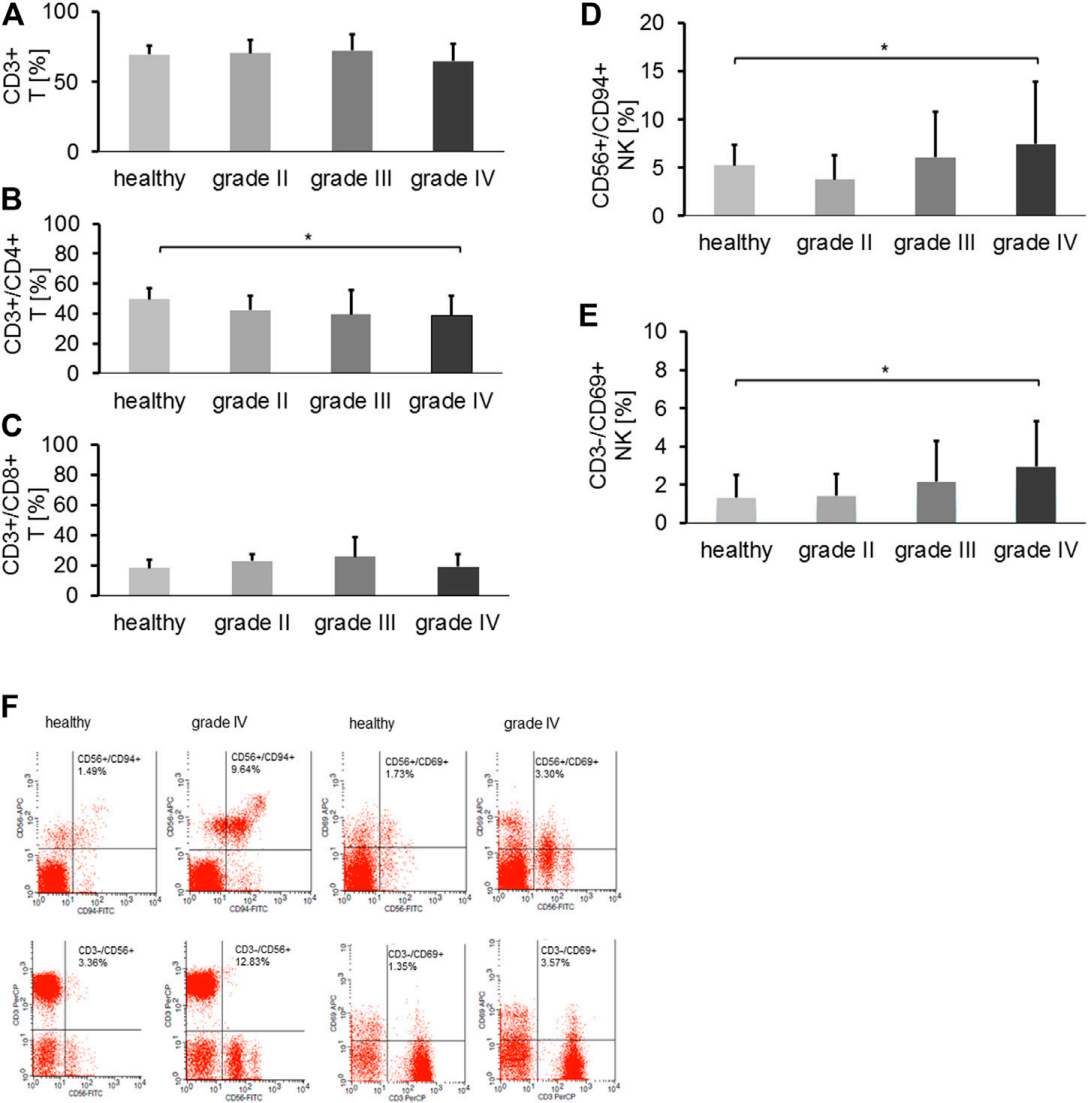
**(A)** The proportion of CD3^+^ T cells in healthy controls (*n* = 15) vs. grade II (*n* = 6), grade III (*n* = 13) and grade IV gliomas (*n* = 56) in the peripheral blood. **(B)** The proportion of CD3+/CD4+ T helper cells in healthy controls (*n* = 15) vs. grade II (*n* = 6), grade III (*n* = 13) and grade IV gliomas (*n* = 56) in the peripheral blood (Tukey; *p < 0.05). **(C)** The proportion of CD3+/CD8+ cytotoxic T cells in healthy controls (*n* = 15) vs. grade II (*n* = 6), grade III (*n* = 13) and grade IV gliomas (*n* = 56) in the peripheral blood. **(D)** The proportion of CD3-/CD94+ NK cells in healthy controls (*n* = 15) vs. grade II (*n* = 6), grade III (*n* = 13) and grade IV gliomas (*n* = 56) in the peripheral blood (*p < 0.05). **(E)** The proportion of CD3-/CD69+ NK cells in healthy controls (*n* = 15) vs. grade II (*n* = 5), grade III (*n* = 13) and grade IV gliomas (*n* = 56) in the peripheral blood (*p < 0.05). **(F)** Representative dot blot analysis of the proportion of CD56+/CD94+ (1.49% vs. 9.64%), CD3-/CD56+ (3.36 vs.12.83%), CD56+/CD69+ (1.73 vs. 3.30%) and CD3-/CD69+ (1.35% vs. 3.57%) NK cells in a healthy control vs. a grade IV glioma patient, respectively.

The proportions of major lymphocyte subpopulations such as CD19^+^ B cells, CD3^+^ T cells, CD3+/CD4+ helper T cells, CD3+/CD8+ cytotoxic T cells, CD4+/CD25+/FoxP3+ and CD8+/CD25+/FoxP3+ Tregs, CD3+/CD56+, CD3+/CD94+, CD3+/NKG2D+ NK-like T cells (NKT), CD56+/CD94+, CD3-/CD56+, CD56+/CD69+, CD3-/CD69+, CD3-/NKG2D+, CD3-/NKp30, and CD3-/NKp40 NK cells were assessed in the peripheral blood of 75 patients with gliomas at first diagnosis (6 grade II, 13 grade III and 56 grade IV) and healthy controls (*n* = 15). The choice of the NK cell receptors was based on the relevance for NK cells to interact with mHsp70 + tumor cells. Neither the percentage of CD19^+^ B cells, CD3+/CD8+ T cells, CD4^+^ and CD8^+^ Tregs nor any of the NK-like T cell subsets differed significantly in patients with gliomas in grade II, III, IV and healthy controls (data not shown). The proportion of CD3^+^ T cells appeared to be slightly reduced in glioblastoma patients at diagnosis compared to healthy controls (Figure 4A). The percentage of CD4^+^ T helper cells (Figure 4B) in grade IV glioma patients at first diagnosis, but not that of CD3+/CD8+ cytotoxic T cells (Figure 4C), was significantly lower than in healthy controls (ANOVA, post-hoc Tukey test, *p < 0.05). Importantly, the proportion of activated NK cell subsets (CD56+/CD94+, CD3-/CD69+) was significantly greater in glioblastoma patients at diagnosis, as compared to healthy controls (Kruskal Wallis test, *p < 0.05) (Figures 4D,E). Jitter plots have been included for T and NK cell markers, including CD3-/NKG2D+, CD3-/NKp30+ and CD3-/NKp46+ NK cell subpopulations, as a Supplementary Figure S2. Representative dot blot analysis of CD56+/CD94+, CD3-/CD56+, CD56+/CD69+ and CD3-/CD69+ NK cell subsets of a healthy individual and a grade IV glioblastoma patient are shown in Figure 4F. The proportion of all NK cell subsets as well as the mean fluorescence intensity values (mfi) of CD94 (69 vs. 43), CD69 (43 vs. 39), and CD56 (70 vs. 40) were higher in a grade IV glioma patient compared to a healthy control, respectively.

### Composition of Lymphocyte Subpopulations in the Peripheral Blood of Healthy Donors After *ex vivo* Stimulation With Hsp70/IL-2 or TKD/IL-2

**FIGURE 5:**
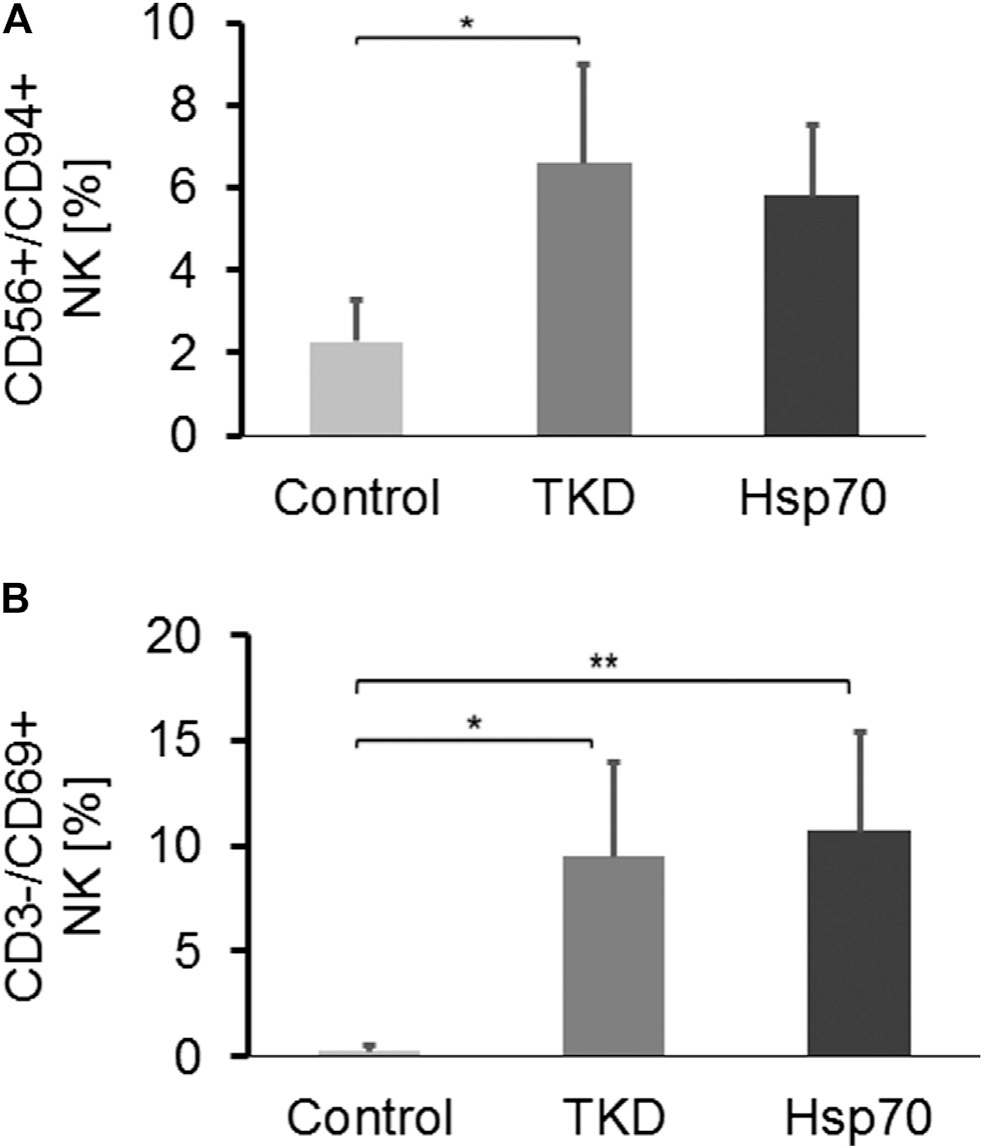
**(A)** The proportion of CD3-/CD94+ NK cells in PBL of unstimulated healthy controls vs. Hsp70-peptide TKD/IL-2 or Hsp70/IL-2 stimulated PBL (*n* = 4) (Tukey; *p < 0.05, **p < 0.01). **(B)** The proportion of CD3-/CD69+ NK cells in PBL of unstimulated healthy controls (*n* = 4) vs. Hsp70-peptide TKD/IL-2 or Hsp70/IL-2 stimulated PBL (*n* = 4).

To mimic potential immunostimulatory effects of circulating Hsp70 in glioblastoma patients, PBL of healthy human donors (*n* = 4) were stimulated *in vitro* either with TKD/IL-2 (TKD) or Hsp70/IL-2 (Hsp70) (Multhoff et al., 1999; Multhoff et al., 2001). Like glioblastoma patients, no significant differences in the composition of CD19^+^ B cells, CD3+/CD4+ and CD3+/CD8+ T cells, NK-like T cells, and Tregs were detected after stimulation (data not shown). However, the proportion of CD56+/CD94+ NK cells, as represented in Figure 5A, was significantly increased after stimulation with TKD/IL-2 (6.6 ± 2.08%, *p < 0.05), as compared to unstimulated PBL (Control, 2.27 ± 0.24%). A similar trend yet not significant, was observed after stimulation with Hsp70/IL-2 (5.81 ± 1.49%). The percentage of CD3-/CD69+ activated NK cells increased significantly after stimulation with TKD/IL-2 (9.48 ± 3.85%, *p < 0.05) and Hsp70/IL-2 (10.69 ± 4.09%, **p < 0.01), as compared to unstimulated PBL (Control, 0.27 ± 0.24%) (Figure 5B).

### Glioblastoma Patients Without Progress Exhibit Elevated Proportions of NK Cells at Diagnosis

A comparison of CD19^+^ B cells, CD3+/CD4+ and CD3+/CD8+ T cells, CD3+/CD4+/CD25+/FoxP3+ and CD3+/CD8+/CD25+/FoxP3+ Tregs and NK-like T cells in glioma patients at diagnosis revealed no significant differences in the prevalence of these cells to that of healthy controls, irrespective of tumor progression at a later time point (data not shown). However, differences were observed with respect to CD56+/CD94+ and CD3-/CD69+ NK cells. Patients with grade III gliomas whose tumors had not progressed after 18 months (*n* = 9) had elevated proportions of CD3-/CD56+ NK cells (8.5 ± 5.3% vs. 6.3 ± 2.8%, data not shown), CD56+/CD94+ NK cells (6.5 ± 5.5% vs. 5.3 ± 3%) and CD3-/CD69+ NK cells (2.4 ± 2.4% vs. 1.6 ± 1.6%) compared to patients whose tumors had progressed (*n* = 3) within 18 months (Supplementary Figure S3A). Similar results were observed in grade IV glioma patients that had not relapsed within 6 months. The proportion of all NK cell subpopulations including CD3-/CD56+, CD56+/CD94+ and activated CD3-/CD69+ NK cells in patients without progression compared to those who progressed within 6 months was higher (11.9 ± 8.9% vs. 9.7 ± 5.1%, 7.1 ± 5.6% vs. 6.9 ± 5.2% and 3.8 ± 3.3% vs. 2.5 ± 1.4%, respectively), although the differences did not reach statistical significance (Supplementary Figure S3B). Compared to healthy individuals (*n* = 15) the proportion of activated NK cells (CD3+/CD69+) in glioblastoma patients who remained progression-free for 6 months (**p < 0.01) was significantly higher (Supplementary Figure S3B). These findings might provide a first hint that an NK cell mediated immunostimulation at an early disease stage is important for a more favorable outcome. Progression was based on Rano criteria for (HGG and LGG) assessed in an interdisciplinary oncological board.

A short-term, low dose glucocorticoid therapy which was given to glioblastoma patients with brain edema before surgery drastically decreases the proportion of CD3-/CD56+ NK cells (13.84 ± 9.79%, *n* = 33 to 8.87 ± 6.53%, *n* = 12, data not shown), CD3-/CD94+ NK cells (8.25 ± 6.25%, *n* = 33 to 5.47 ± 4.41%, *n* = 12) and CD3-/CD69+ NK cells (4.56 ± 5.37%, *n* = 33 to 2.86 ± 2.22%, *n* = 12) (Supplementary Figure S3C).

Kaplan–Meier curves showing an association of progression-free survival probability with an elevated frequency of CD3-/CD94+ and CD3-/CD56+ NK cells were performed to evaluate the impact of NK cells in prognosis of glioblastoma patients. As illustrated in Supplementary Figure 4A (CD3-/CD94+) and Figure 4B (CD3-/CD56+) glioblastoma patients with higher NK cell counts (>5% and >7.5%, respectively) have a trend toward a more favorable progression-free survival—which also reflects the OS in glioblastoma patients—than patients with lower NK cell counts (*p* = 0.05).

## Discussion

In 2018 the Nobel prize in Physiology and Medicine was awarded to James P. Allison and Tasuku Honjo for their discovery of immunoregulatory mechanisms mediated by immune checkpoint inhibitors (Sharma and Allison, 2020), but already years before the interest in immunotherapeutic clinical approaches including immune checkpoint inhibitor blockades (Postow et al., 2015) and genetically engineered chimeric antigen receptor (CAR)-T/NK cell therapies has attracted the attention of many immunologists (Guo, 2018; Akhavan et al., 2019; Brown et al., 2019; Kruger et al., 2019). However, despite promising results in a number of cancer entities, a significant proportion of patients, including those with brain tumors, do not profit from these therapies due to immunosuppressive mechanisms of the tumor and its microenvironment (Huang et al., 2017; Goswami et al., 2020). Therefore, a better understanding of immunosuppressive and immunostimulatory effects in patients with gliomas is urgently needed. In this study, we aimed to identify chaperone-based and immune-related (lymphocyte subpopulations) biomarkers for predicting of prognosis of glioblastoma. Depending on its subcellular or extracellular localization, Hsp70 fulfills different tasks. Extracellular and membrane-bound Hsp70 exert immunostimulatory effects on NK cells, whereas high cytosolic Hsp70 levels interfere with apoptotic pathways and thereby mediate therapy resistance (Goswami et al., 2020). Therefore, intra-, membrane-bound and extracellular Hsp70 levels were measured for the first time in patients with gliomas in different stages comparatively. As expected, high cytosolic Hsp70 levels are associated with a more aggressive and advanced glioma type. A membrane Hsp70 positivity was found in all glioma types. Significantly elevated extracellular Hsp70 levels that might act as danger associated molecular patterns (DAMPs) to stimulate NK cells were found in large, highly necrotic grade IV glioblastomas. Data of clinical trials using *ex vivo* Hsp70 peptide and IL-2 stimulated, autologous NK cells to treat patients with advanced colon and lung cancers after radiochemotherapy (Krause et al., 2004; Multhoff et al., 2020) reveal that the cytolytic activity of patient-derived, anergic NK cells could be re-stimulated by Hsp70 peptide and IL-2, but not by IL-2 alone (Krause et al., 2004). Favorable clinical responses in a randomized phase II clinical trial in patients with advanced NSCLC could be attributed to an increased prevalence of activated CD94^+^ NK cells (Multhoff et al., 2020) recognizing mHsp70+ tumor cells (Gross et al., 2003). The increased cytolytic activity of Hsp70-activated NK cells can be blocked either by a CD94 specific blocking antibody for NK cells or by an Hsp70 specific antibody for blocking mHsp70 on tumor cells (Krause et al., 2004). Previously, we demonstrate that only MACS purified CD3^−^ NK cells (>97%), but not CD3^+^ T or γ/δ T cells are able to recognize mHsp70+ tumor cells after stimulation with Hsp70 protein or TKD and IL-2 (Gross et al., 2003). Therefore, other effector cell populations can be excluded for the recognition of mHsp70+ tumor cells. The lysis of mHsp70+ tumor cells by NK cells could be attributed to granzyme B (Gross et al., 2003). Following binding and uptake, granzyme B is able to efficiently kill mHsp70+ tumor cells (Shevtsov et al., 2019). The production of granzyme B in NK cells of patients with solid tumors (Bücklein et al., 2016) is frequently impaired, but can be reconstituted by a stimulation with IL-2 and Hsp70 protein or TKD (Multhoff et al., 1999; Multhoff et al., 2001; Pockley et al., 2020). Therefore, the proportion of activated CD94^+^ NK cells was studied together with extracellular and membrane-bound Hsp70 levels in tumor cells.

In contrast to non-transformed control brain tissues, nearly all highly malignant tumor cells exhibit an upregulated cytosolic Hsp70 expression and a mHsp70 positivity that contributes to therapy resistance, but also serves as a target for NK cells (Ciocca and Calderwood, 2005; Murakami et al., 2015). In line with these results, an upregulated nuclear and cytosolic Hsp70 expression was predominantly found in high grade gliomas (Beaman et al., 2014; Ceccarelli et al., 2016), whereas low grade gliomas show a weaker and purely nuclear staining pattern. Due to the limited number of patients with low grade gliomas, future studies with larger patient cohorts are necessary to assess more detailed the subcellular localization of Hsp70.

Compared to healthy controls, significantly elevated serum levels of free Hsp70 were observed in glioblastoma patients with large necrotic tumor areas, but not in low grade gliomas. From this we conclude that extracellular Hsp70 predominantly originates from dying tumor cells (Breuninger et al., 2014). An intact blood-brain-barrier in patients with low grade gliomas might hinder the release of Hsp70 into the circulation of these patients. Liposomal Hsp70 levels (Breuninger et al., 2014) predominantly originating from exosomes (Gastpar et al., 2005) which are assumed to be actively released from viable tumor cells, were higher in grade IV compared to lower grade gliomas, but were not found to be increased in patients with necrotic tumors.

An improved OS of glioblastoma patients was associated with Hsp70 serum levels below a threshold of 3.5 ng/ml which is typically found in healthy individuals. Although the data did not reach statistical significance, these findings support the hypothesis that the aggressiveness of glioblastomas is associated with higher Hsp70 levels.

Extracellular Hsp70 either free or in exosomes (Multhoff et al., 1999; Gastpar et al., 2005; Pockley et al., 2020) together with other DAMPs derived from necrotic tumor cells can stimulate the release of pro-inflammatory cytokines including IL-2. We have previously established that Hsp70 in combination with IL-2 stimulates NK cell-mediated immune responses mediated by activatory receptors such as CD94/NKG2C can be further enhanced by immune checkpoint inhibition (Gross et al., 2003; Shevtsov et al., 2019). To test the hypothesis of an Hsp70-mediated immunostimulation of CD56+/CD94+/CD69+ NK cells, PBLs from healthy donors were stimulated with recombinant Hsp70 or the TKD Hsp70-peptide together with low dose IL-2. This *in vitro* stimulation resulted in a similar upregulation of the frequency and density of activatory NK cell receptors such as CD94^+^ and CD69^+^ on NK cells to that observed in glioblastoma patients. An upregulated CD94 expression on NK cells has been shown to correlate with an elevated cytolytic activity against mHsp70+ tumor cells *in vitro* and in clinical trials (Multhoff et al., 1999; Krause et al., 2004; Kokowski et al., 2019; Multhoff et al., 2020). Although the measured Hsp70 serum levels of glioblastoma patients are too low for an efficient NK cell stimulation it cannot be ruled out that locally increased Hsp70 levels in glioblastoma patients might activate the NK cell activity.

In line with the literature, patients with high grade gliomas in our study exhibit a decreased frequency of CD3+/CD4+ T cells (Mcvicar et al., 1992; Fecci et al., 2007). However, the value of tumor-infiltrating NK cells as a biomarker for gliomas is not completely understood. Although one study denies an involvement, another study indicates that NK cell infiltration into the tumor microenvironment is more common in high grade than in low grade gliomas (Stevens et al., 1988; Yang et al., 2011). Due to the KIR2DS400101 allele being associated with the capacity to kill glioblastoma cells, NK cell-based immunotherapies are discussed as an additional treatment option in combination with surgery and radiochemotherapy (Gross et al., 2003; Castriconi et al., 2009; Dominguez-Valentin et al., 2016). Moreover, targeted CAR-NK cell-based immunotherapies are presently tested in preclinical and clinical studies of glioblastoma (Zhang et al., 2016; Murakami et al., 2018; Burger et al., 2019). However, cancer-initiating cells (CICs) escaping immune recognition, elevated Treg, M2 macrophage and MDSC counts in the tumor microenvironment, immunosuppressive factors (i.e., IL-1, TGF-β, IL-10, arginase I), apoptosis-inducers (i.e., CD95, CD70), tumor hypoxia and immune checkpoint inhibitors are major challenges also for NK cell-based immunotherapies (Nduom et al., 2015; Maccalli et al., 2017; Multhoff and Vaupel, 2020). Future studies are necessary to elucidate the capacity of NK and CAR-NK cells to overcome these immunosuppressive conditions in glioblastoma. Our data provide a first hint that an increased prevalence of activated NK cells at diagnosis are associated with a more favorable outcome in glioblastoma patients. Our preliminary data indicate that a pre-surgical treatment with glucocorticoids can reduce the prevalence of these NK cells. Since long-term glucocorticoid therapy has been shown to exert unfavorable effects on the survival of glioblastoma patients treated with RT and/or CT (Petrelli et al., 2020) and exert immunosuppressive activities in combination with immune checkpoint inhibitor therapies (Kelly and Gilbert, 2020) their use should be considered with caution.

## Conclusion

We demonstrate that Hsp70 is presented on the plasma membrane of all gliomas as a potential target for NK cells and a strong nuclear and cytosolic Hsp70 expression is associated with high grade gliomas. The present study provides first evidence that low Hsp70 levels and elevated proportions of activated CD94+/CD69+ NK cells able to recognize mHsp70+ tumor cells (Multhoff et al., 1999) in glioblastoma patients might favor a beneficial clinical outcome. However, due to the relatively low number of patients with low grade gliomas the results need to be confirmed in larger patient cohorts.

## Data Availability

The raw data supporting the conclusion of this article will be made available by the authors, without undue reservation.
