# Supplementary material for: Potential Role of Hsp70 and Activated NK Cells for Prediction of Prognosis in Glioblastoma Patients
Source: Front Mol Biosci. 2021 May 17;8:669366. doi: 10.3389/fmolb.2021.669366 (PMC8165168; doi:10.3389/fmolb.2021.669366)
Supplement: Supplementary file 1 [file Presentation1.PPTX]

## Slide 1
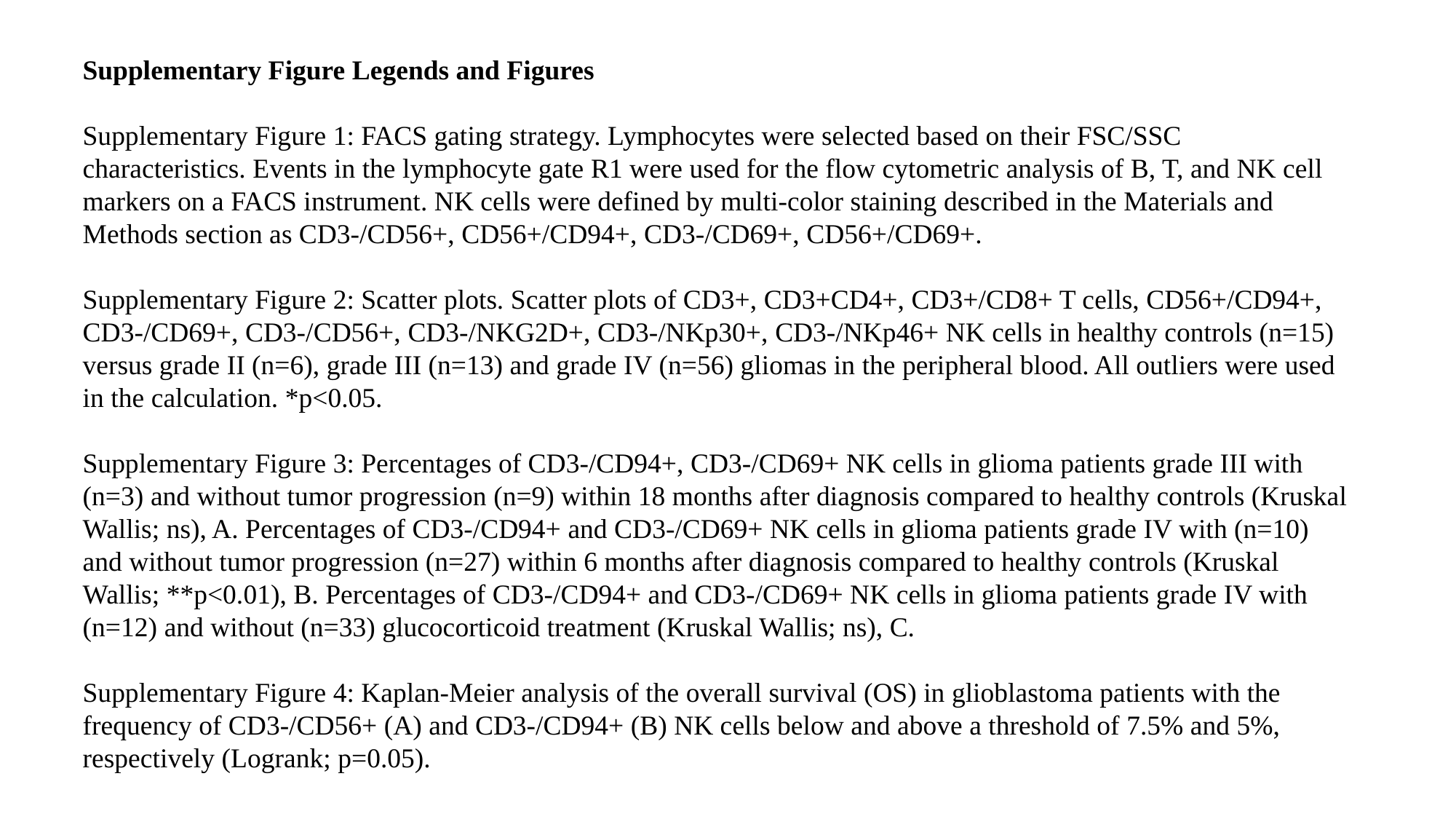

Supplementary Figure Legends and Figures
Supplementary Figure 1: FACS gating strategy. Lymphocytes were selected based on their FSC/SSC characteristics. Events in the lymphocyte gate R1 were used for the flow cytometric analysis of B, T, and NK cell markers on a FACS instrument. NK cells were defined by multi-color staining described in the Materials and Methods section as CD3-/CD56+, CD56+/CD94+, CD3-/CD69+, CD56+/CD69+.
Supplementary Figure 2: Scatter plots. Scatter plots of CD3+, CD3+CD4+, CD3+/CD8+ T cells, CD56+/CD94+, CD3-/CD69+, CD3-/CD56+, CD3-/NKG2D+, CD3-/NKp30+, CD3-/NKp46+ NK cells in healthy controls (n=15) versus grade II (n=6), grade III (n=13) and grade IV (n=56) gliomas in the peripheral blood. All outliers were used in the calculation. *p<0.05.
Supplementary Figure 3: Percentages of CD3-/CD94+, CD3-/CD69+ NK cells in glioma patients grade III with (n=3) and without tumor progression (n=9) within 18 months after diagnosis compared to healthy controls (Kruskal Wallis; ns), A. Percentages of CD3-/CD94+ and CD3-/CD69+ NK cells in glioma patients grade IV with (n=10) and without tumor progression (n=27) within 6 months after diagnosis compared to healthy controls (Kruskal Wallis; **p<0.01), B. Percentages of CD3-/CD94+ and CD3-/CD69+ NK cells in glioma patients grade IV with (n=12) and without (n=33) glucocorticoid treatment (Kruskal Wallis; ns), C.
Supplementary Figure 4: Kaplan-Meier analysis of the overall survival (OS) in glioblastoma patients with the frequency of CD3-/CD56+ (A) and CD3-/CD94+ (B) NK cells below and above a threshold of 7.5% and 5%, respectively (Logrank; p=0.05).

## Slide 2
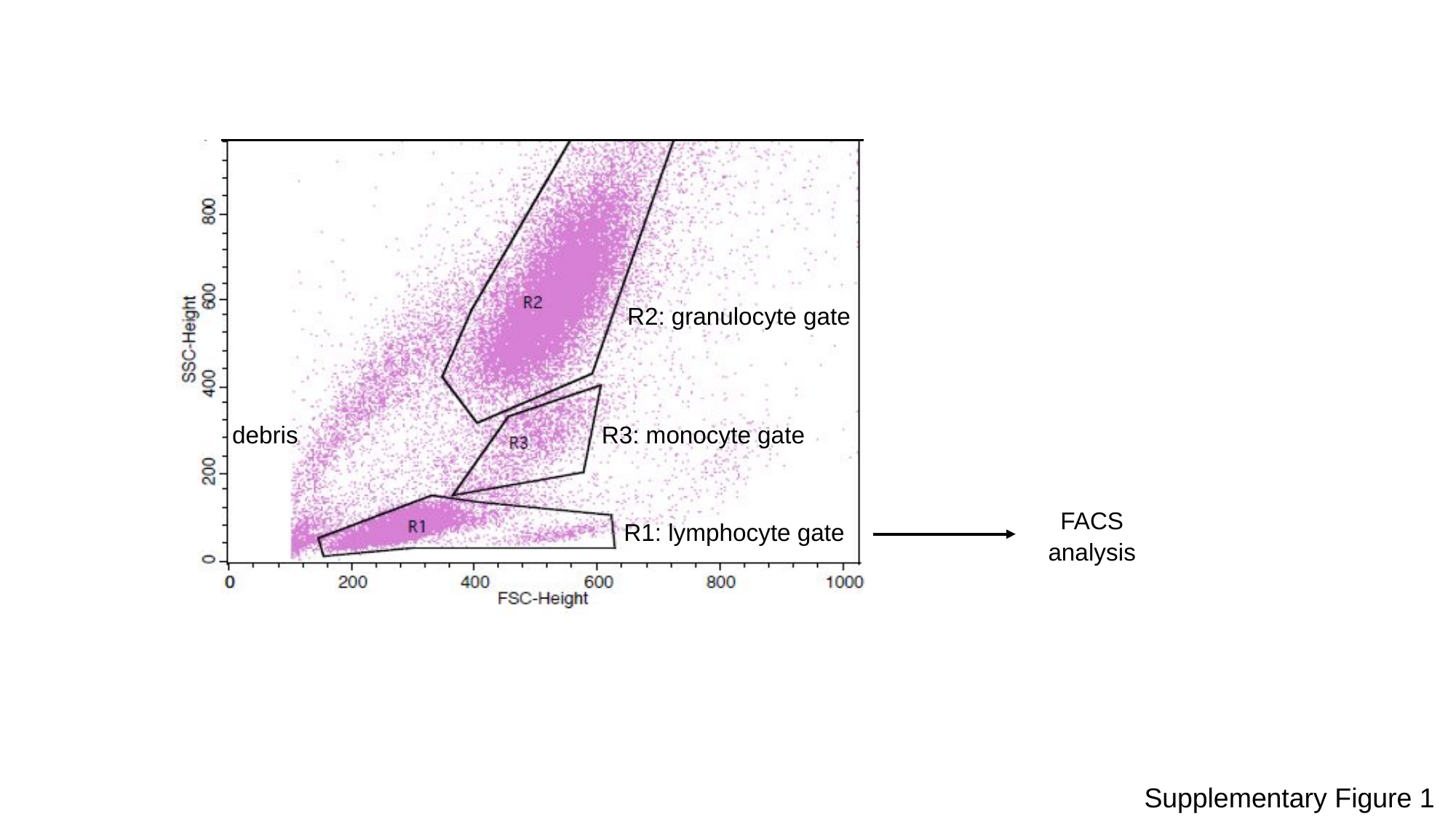

R2: granulocyte gate
debris
R3: monocyte gate
R1: lymphocyte gate
FACS analysis
Supplementary Figure 1

## Slide 3
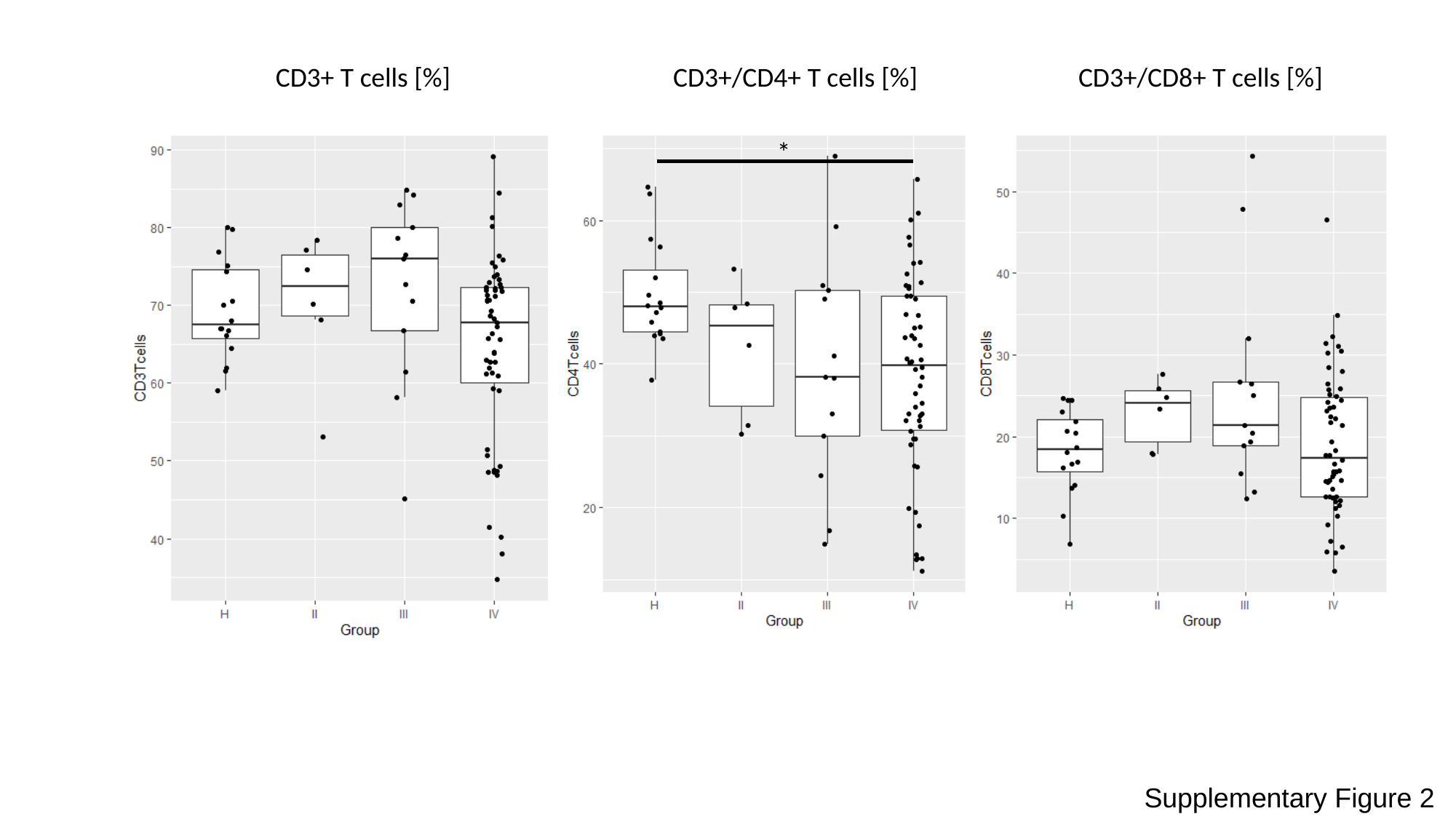

CD3+ T cells [%] CD3+/CD4+ T cells [%] CD3+/CD8+ T cells [%]
*
Supplementary Figure 2

## Slide 4
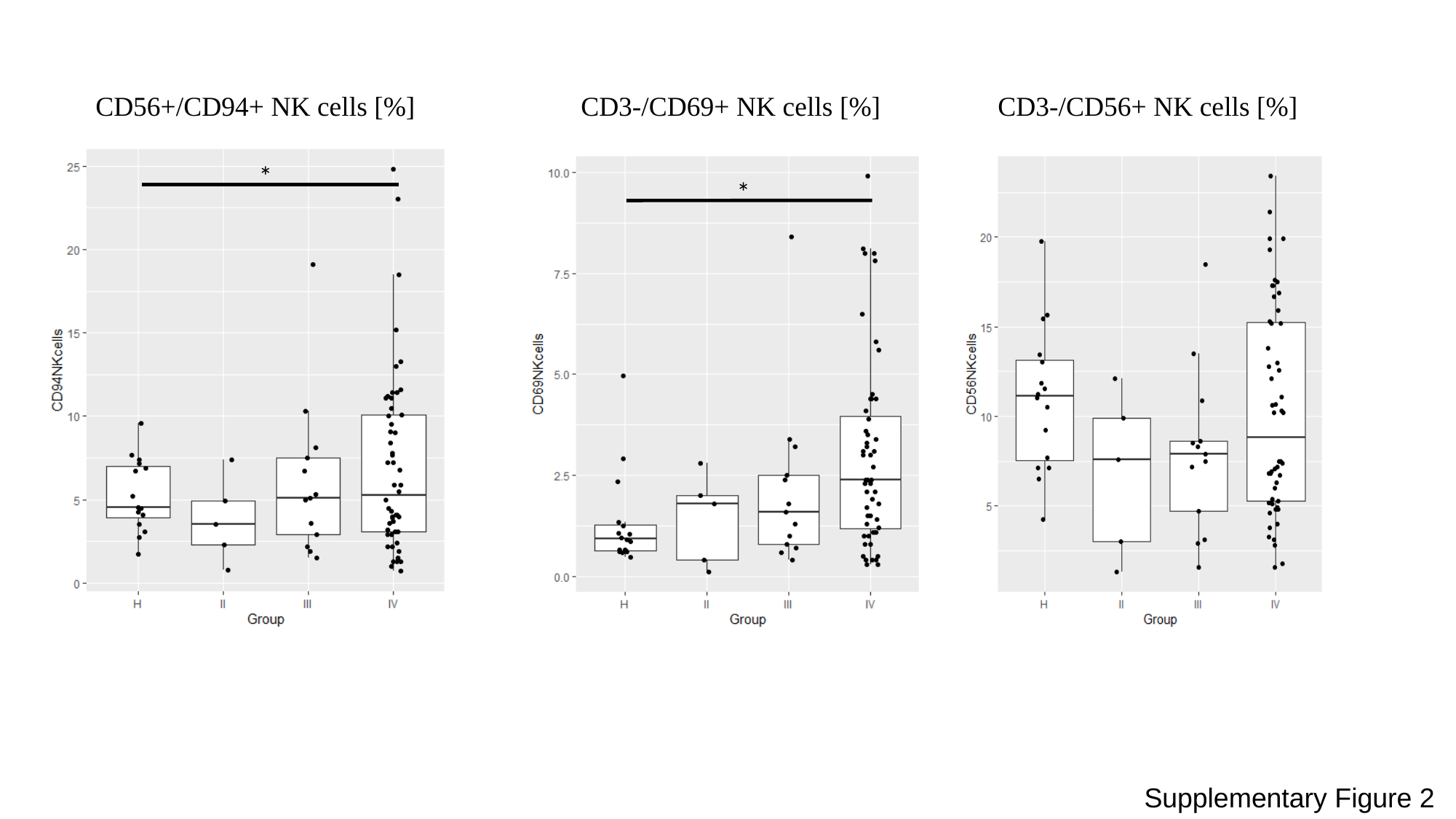

CD56+/CD94+ NK cells [%] CD3-/CD69+ NK cells [%] CD3-/CD56+ NK cells [%]
*
*
Supplementary Figure 2

## Slide 5
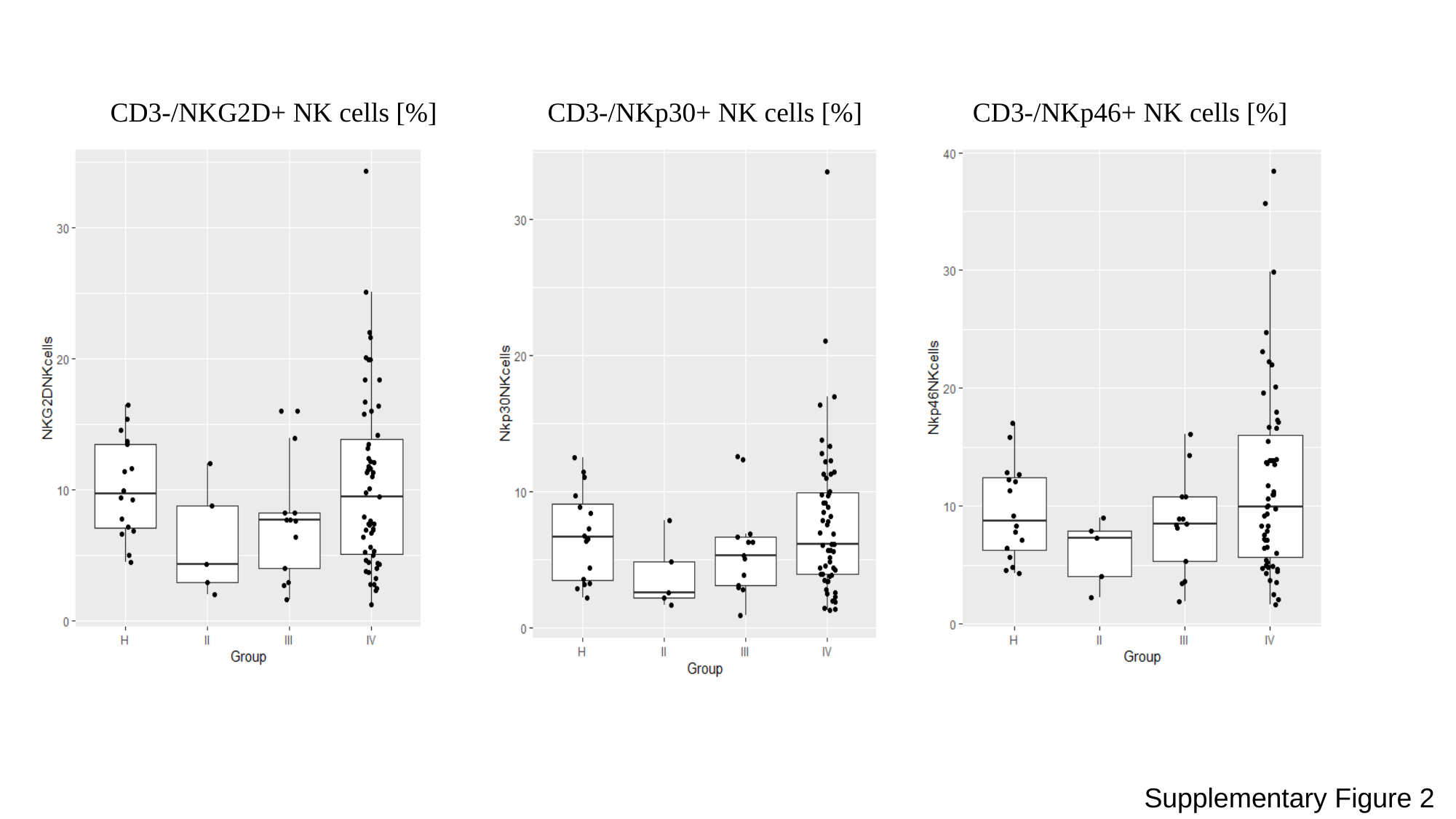

CD3-/NKG2D+ NK cells [%] CD3-/NKp30+ NK cells [%] CD3-/NKp46+ NK cells [%]
Supplementary Figure 2

## Slide 6
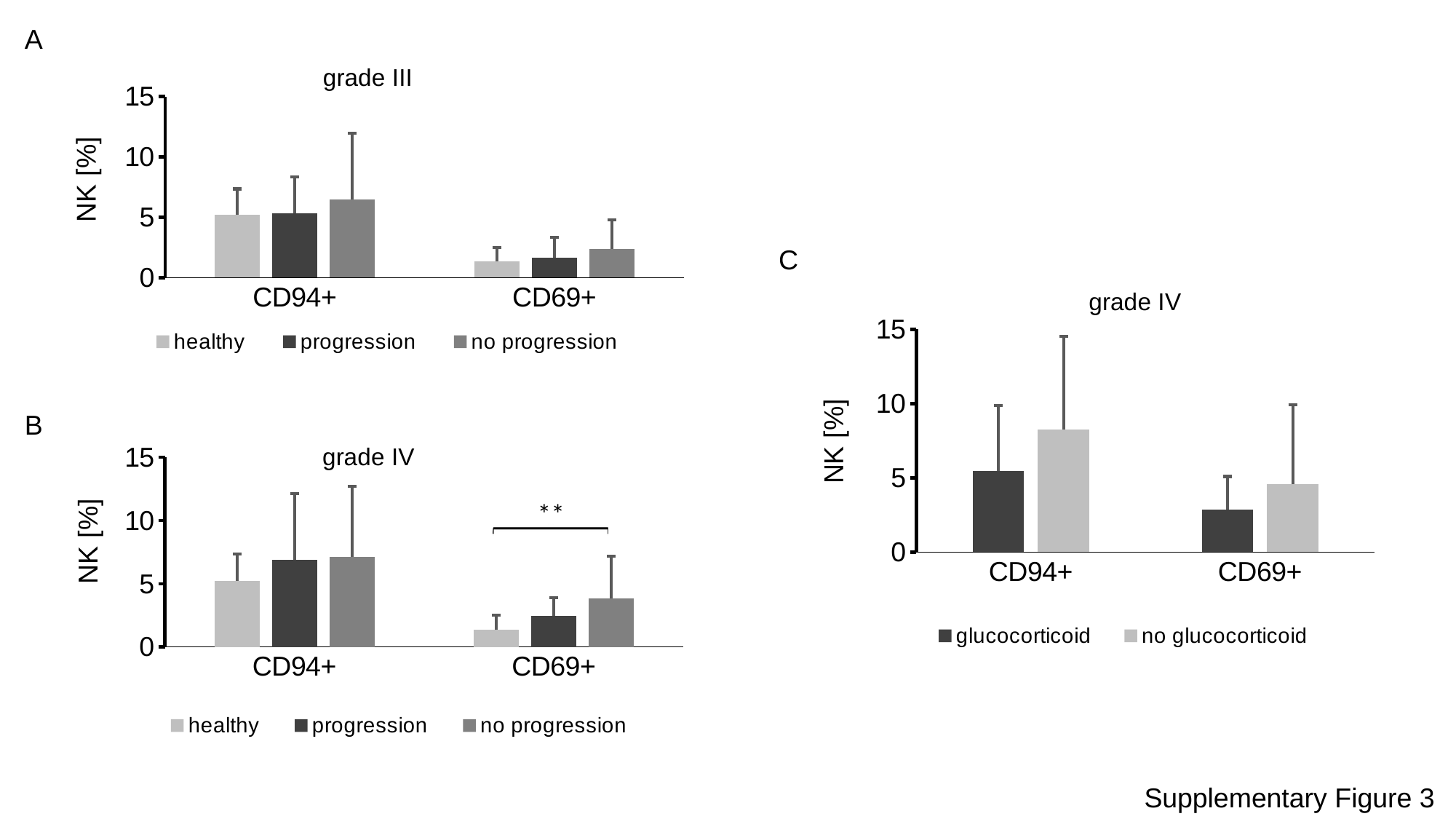

A
### Chart: grade III
| Category | healthy | progression | no progression |
|---|---|---|---|
| CD94+ | 5.218124999999999 | 5.343333333333334 | 6.4577777777777765 |
| CD69+ | 1.3287499999999999 | 1.6633333333333333 | 2.36 |C
### Chart: grade IV
| Category | glucocorticoid | no glucocorticoid |
|---|---|---|
| CD94+ | 5.470833333333334 | 8.253030303030302 |
| CD69+ | 2.864166666666667 | 4.555 |B
### Chart: grade IV
| Category | healthy | progression | no progression |
|---|---|---|---|
| CD94+ | 5.218124999999999 | 6.894 | 7.090357142857143 |
| CD69+ | 1.3287499999999999 | 2.467777777777778 | 3.823571428571429 |
**
Supplementary Figure 3

## Slide 7
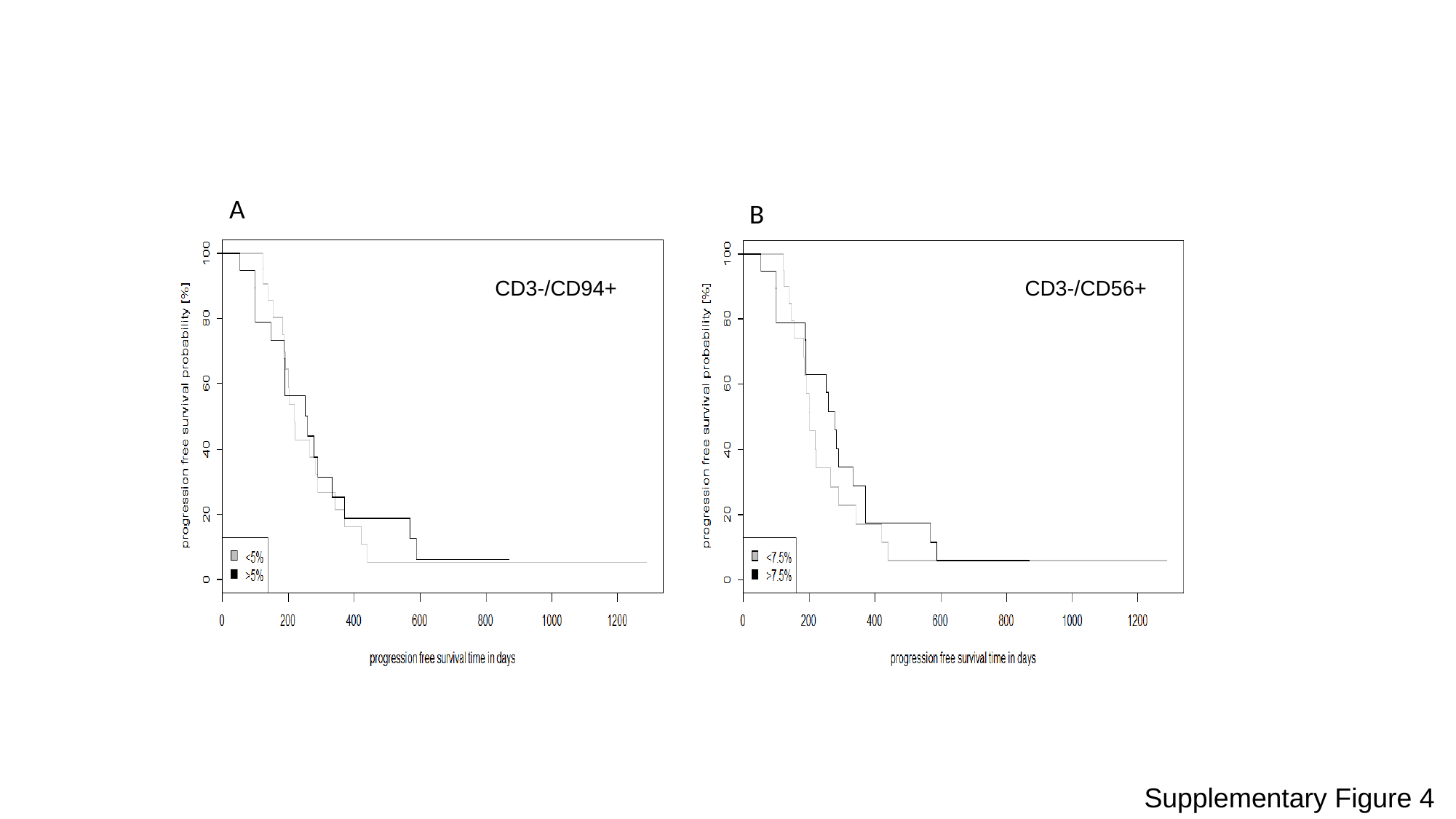

A
B
CD3-/CD94+
CD3-/CD56+
Supplementary Figure 4
